# Fire activity as measured by burned area reveals weak effects of ENSO in China

**DOI:** 10.1038/s41467-022-32013-9

**Published:** 2022-07-28

**Authors:** Víctor Resco de Dios, Yinan Yao, Àngel Cunill Camprubí, Matthias M. Boer

**Affiliations:** 1 School of Life Science and Engineering, Southwest University of Science and Technology, Mianyang, China; 2 Department of Crop and Forest Sciences, University of Lleida, Lleida, Spain; 3 Joint Research Unit CTFC-AGROTECNIO-CERCA Center, Lleida, Spain; 4 Hawkesbury Institute for the Environment, Western Sydney University, Richmond, NSW Australia

**Keywords:** Natural hazards, Fire ecology

**arising from** Fang et al. *Nature Communications* 10.1038/s41467-021-21988-6 (2021)

Wildfire activity is being perceived as an increasing problem in many areas worldwide, but its definition remains elusive and difficult to quantify at large spatial scales. A recent study, based on a comprehensive database of fire occurrences (Wildfire Atlas of China, WFAC)^1^, concluded that the majority of fire activity in China is concentrated in its tropical and subtropical forests and significantly driven by ENSO. Here we demonstrate that a very different picture emerges when wildfire activity is assessed from burned area instead. Using fire occurrences as the sole indicator of fire activity, particularly to compare regions with markedly different fire regimes, may lead to incomplete descriptions of fire activity with potential implications for fire policy recommendations.

## Burned area fraction across China’s subtropical forests is low nationally and globally

**Fig. 1:**
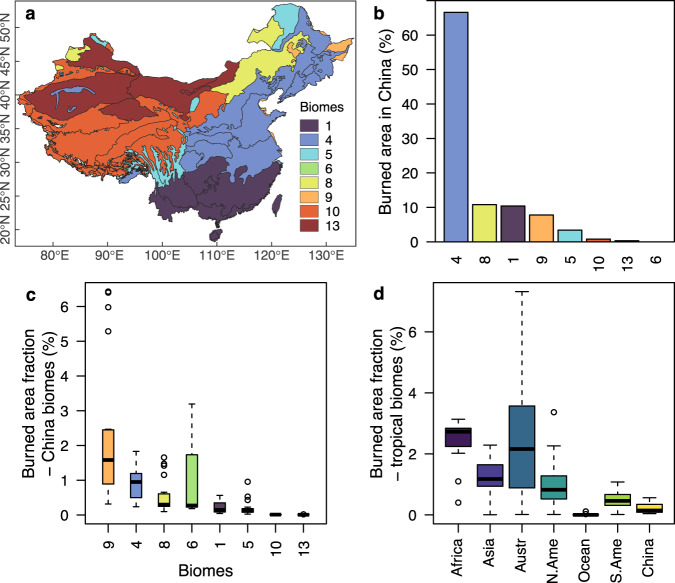
Burned area in China and in tropical biomes worldwide. **a** Map with China’s biomes. Black lines delineate ecoregions^14^. **b** Burned area distribution across the biomes of China from the MCD64A1 burned area product (GlobFire Database, 2001-2020)^2^. **c** Fraction of annual burned area, relative to the total area, for each biome in China. **d** Fraction of burned area across tropical forests in different continents from Boer et al.^8^ and in China. Boxplots in **c** and **d** indicate the median value, with hinges showing the first and third quartiles and the whiskers extending up to 1.5 times the interquartile range. Biome delineation from Dinerstein et al.^14^: 1, tropical and subtropical moist broadleaf forests; 4, temperate broadleaf and mixed forests; 5, temperate conifer forests; 6, boreal forests/taiga; 8, Temperate Grasslands, Savannas & Shrublands; 9, Flooded Grasslands & Savannas; 10, Montane Grasslands & Shrublands; 13, Deserts & Xeric Shrublands.

We assessed patterns in the burned area during 2001–2020 from the MODIS Collection 6 (C6) MCD64A1 burned area product, using previously curated and compiled datasets^2^. We observed that only 10% of the burned area in China occurred within the tropical and subtropical moist broadleaf forests biome of China (Fig. 1a, b, subtropical forests hereafter). The vast majority of the burned area (67%) occurred in the temperate broadleaf and mixed forests biome, followed by the temperate grasslands, savannas and shrublands biome (11%). Fang et al.^1^ concluded that wildfire activity was concentrated in the subtropical forests because this is where 84% of all fire events occurred^1^, but here we show that they only represent 10% of the total burned area (Fig. 1a, b). Even after removing the effect of agricultural fires (particularly common in northeast China^3^), the burned area outside the subtropical biome remains at 74% of the total burned area (Supplementary Fig. 1). Burned area is a more comprehensive measure of continental or regional fire activity than fire occurrence, amongst others, because fire-size distributions differ across biomes^4^ and because the inverse of burned area approximates the fire cycle (that is, the number of years necessary to burn entirely a particular region).

We additionally examined the fraction of annual burned area, relative to the total area occupied by each biome. The annual fraction of burned area in Chinese subtropical forests was 0.2%, which is three times lower than the national average of 0.7% (Fig. 1c). The fraction of median annual burned area ranged from 0.006% for the desert and xeric shrublands biome to 1.6% for the flooded grasslands and savannas biome. We note that the MCD64A1 burned area product underestimates burned area, particularly in tropical and subtropical forests where cloudy days are common^5^. However, this effect is most important for small fires, while the burned area is primarily determined by large fires, where MODIS accuracy increases^6^. The comparatively small fraction of burned area in subtropical forests is consistent with the pyrogeographic principle that fire activity in moist tropical forests is putatively limited by its high humidity and fuel moisture^7^.

Fang et al.^1^ claimed that fire activity in China’s subtropical forests is higher than in other subtropical forest regions. Recent estimates of the fraction of annual burned area across subtropical forests globally^8^ report a variation that ranges from 0.01% in Oceania up to 2.5% in Africa, with a global mean of 1.1% (Fig. 1d). The annual fraction of burned area in China’s subtropical forests is five times lower (0.2% as previously stated) than the global average (Fig. 1d) and only higher than in Oceania’s tropical forests. Consequently, China’s tropical forests are amongst the lowest globally in terms of the annual fraction of burned area.

## No dipole in burned area between eastern and western subtropical forests

**Fig. 2:**
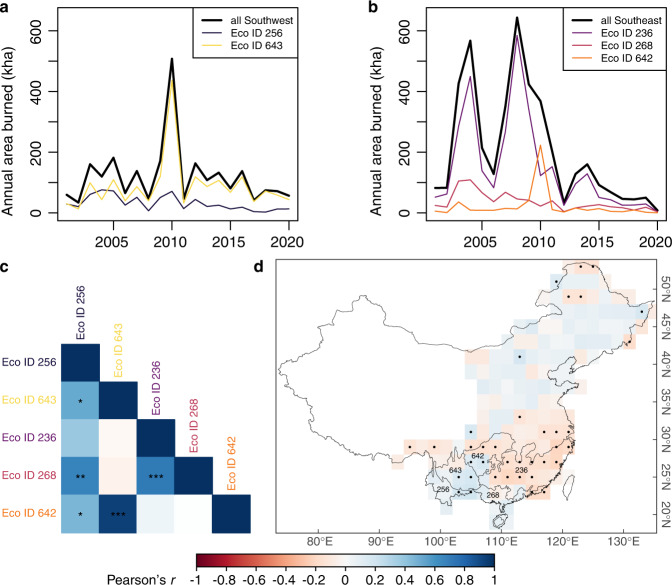
There is no dipole in the pattern of burned area between eastern and western tropical forests in China. We examined annual patterns of annual area burned in **a** southwest China and its ecoregions, and in **b** southeast China and its ecoregions. **c** Pearson correlations in annual burned area across ecoregions (one, two or three asterisks indicate significant correlations at *P* < 0.05, <0.01 and <0.0001 respectively) were positive within eastern and within western ecoregions, and either positive or not-significant across eastern and western ecoregions. **d** We correlated monthly burned area and ENSO 3.4 SST index at different lags (0, 3, 4, 5, 6 and 12 months) and show the highest correlation in absolute terms (significant correlations are indicated by a dot). Correlations in **c** and **d** are indicated by the color. Ecoregion IDs from Dinerstein et al.^14^: 236, Jian Nan subtropical evergreen forests; 256, Northern Indochina subtropical forests; 268, South China-Vietnam subtropical evergreen forests; 642, Guizhou Plateau broadleaf and mixed forests; 643 Yunnan Plateau subtropical evergreen forests.

Under the assumption that the bulk of fire activity occurs in subtropical China, the authors reported a dipole between fire occurrence in subtropical forest regions of southeastern and southwestern China. We tested whether this dipole also occurred for the burned area by examining the correlations between annual area burned across eastern and western subtropical ecoregions. If a dipole in fire activity exists, we should observe a negative east-west trend. However, correlations across eastern and western subtropical ecoregions were either non-significant (*P* > 0.1) or positive (*P* < 0.005 and *r* > 0.5; Fig. 2a–c), which is inconsistent with the notion of a dipole in fire activity.

## ENSO effects on burned area weak or nonsignificant

Fang et al.^1^ further claimed that the mechanism driving the dipole lies in the differential effect of ENSO over climate across the nation and, consequently, fire activity. Although we did not observe the dipole in the burned area (Fig. 2a–c) we did examine whether monthly area burned correlated with El Niño 3.4 SST index at different lags (lags 0, 3, 4, 5, 6 and 12 months), following previous approaches appropriate for time series analyses^9^, and after dividing China in 2° × 2° grid cells^1^. There was a tendency for positive correlations to dominate over western subtropical forests and for negative correlations to dominate in eastern subtropical forests (Fig. 2d). However, the effect of ENSO over burned area was non-significant for over 53% of the subtropical biome and, when significant, the effect was rather weak (r<|0.25|). The effect of ENSO over burned area thus ranges between nonsignificant and weak.

## Fire suppression does not affect wildfire occurrence

Mixing fire occurrence with wildfire activity is problematic also when trying to draw policy conclusions. Fang et al.^1^ examined the temporal pattern of fire numbers between 2005-18 and concluded that the application of a fire suppression policy after 1987 has contributed to decreases in fire occurrences after 2007. However, fire suppression is an effort to mitigate the results of a fire once it has started^10^. Consequently, fire suppression strictly affects the burned area, and not fire occurrence. Other aspects associated with fire planning, like awareness campaigns or fire bans, may act on fire occurrence. However, any relationship between fire occurrence and fire suppression will necessarily be artefactual because the latter does not affect the former.

We acknowledge that part of the discrepancy with Fang et al.^1^ may lie in the different scales used in these analyses. However, fire activity is a term that currently lacks a rigorous definition and should be used with caution. Fire occurrence depends primarily on the number of ignitions (along with other factors affecting fire detection such as climate, topography or vegetation), which, in turn, results from human activity^1^ and, in some areas, lightning^11^. Using fire occurrence as an indicator for fire activity is particularly problematic when comparing multiple biomes that show marked differences in fire regime, as we demonstrate here. Additionally, ENSO and fire suppression may both affect burned area, but there is currently no mechanism that can explain a mechanistic link between either of these processes and the number of fire events. Consequently, fire occurrence should not be used as a sole metric of fire activity.

We additionally note that burned area is not necessarily a reliable metric of fire impacts on ecosystems and society. Significant variation in severity and intensity may occur within a fire perimeter^12^. Additionally, damage to people and property are not captured by this metric^13^. While we caution against the use of a single metric to evaluate fire activity, we hope to have demonstrated that using fire occurrence alone is particularly problematic, and that the picture it paints is rather unrealistic.

## Supplementary information

Supplementary Information

## Data Availability

All data sources are cited within the text.

## Code availability

The computer code used to generate the figures will be made available upon request.
