# Supplementary material for: Fire activity as measured by burned area reveals weak effects of ENSO in China
Source: Nat Commun. 2022 Jul 28;13:4316. doi: 10.1038/s41467-022-32013-9 (PMC9334383; doi:10.1038/s41467-022-32013-9)
Supplement: Supplementary file 1 — Supplementary Information [file 41467_2022_32013_MOESM1_ESM.pdf]

Supplementary Information for “Measuring fire activity from burned area reveals weak effects of ENSO in China” by Resco de Dios et al.

Supplementary Information

Measuring fire activity from burned area reveals weak effects of ENSO in China

Víctor Resco de Dios[1,2,3,*], Yinan Yao[1], Àngel Cunill Camprubí[3], Matthias Boer[4]

[1] School of Life Science and Engineering, Southwest University of Science and Technology, Mianyang, China

[2] Department of Crop and Forest Sciences, University of Lleida, Lleida, Spain

[3] Joint Research Unit CTFC-AGROTECNIO-CERCA Center, Lleida, Spain

[4] Hawkesbury Institute for the Environment, Western Sydney University, Richmond, NSW, Australia

* Corresponding author (victor.resco@udl.cat)

Matters Arising from Fang. et al. ENSO modulates wildfire activity in China. *Nat. Comm.* **12**, 1764 (2021).

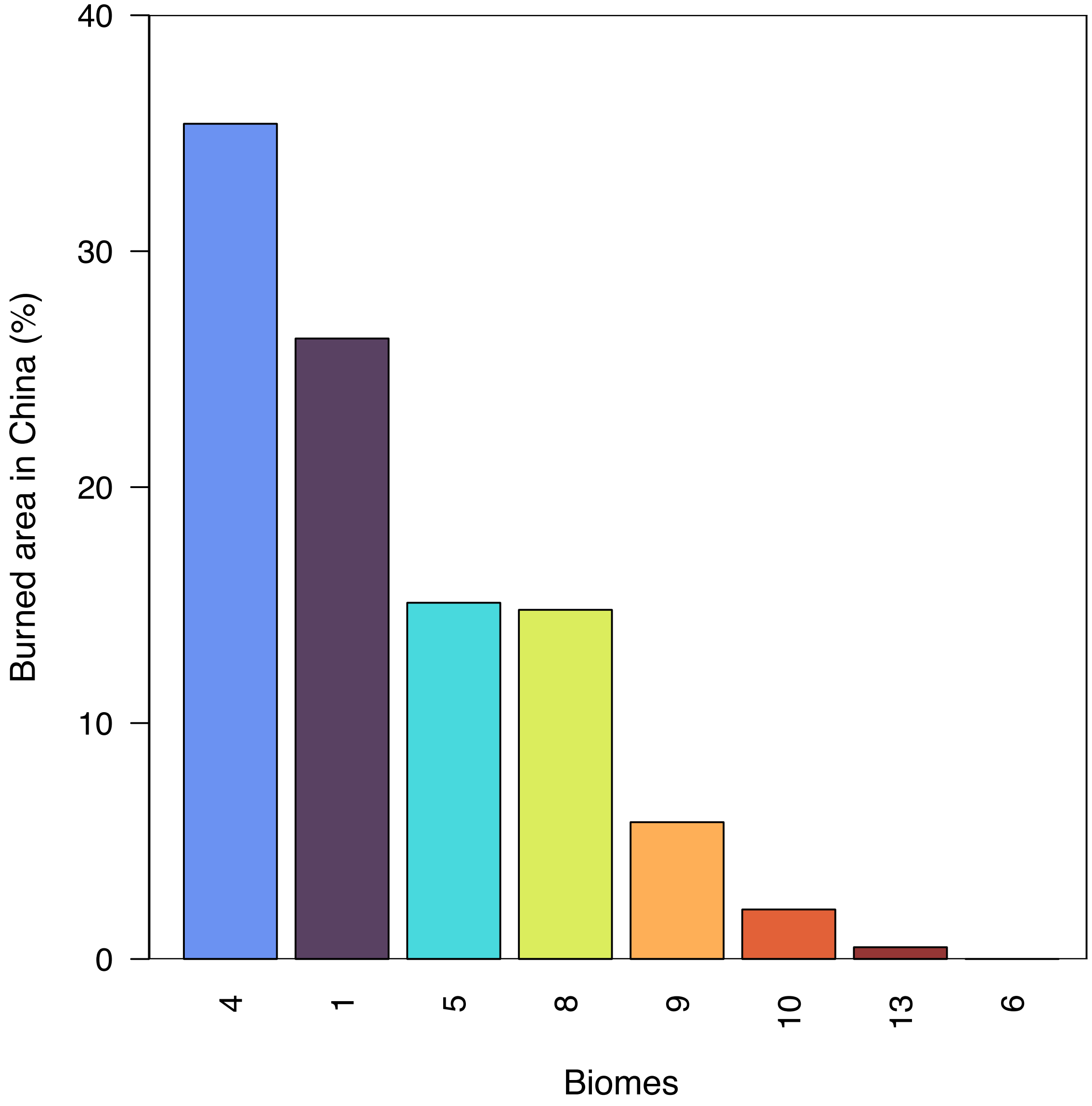

Supplementary Figure 1 | Burned area within the subtropical forests biome of China only represents 26% of total burned area after excluding agricultural fires. Burned area distribution across the biomes of China from the MCD64A1 burned area product (GlobFire Database, 2001-2020)[1], after excluding burned area on agricultural land using the CCI Land Cover Dataset[2]. Biome definitions from Dinerstein et al [3]: 1, tropical and subtropical moist broadleaf forests; 4, temperate broadleaf and mixed forests; 5, temperate conifer forests; 6,

boreal forests/taiga; 8, Temperate Grasslands, Savannas & Shrublands; 9, Flooded Grasslands & Savannas; 10, Montane Grasslands & Shrublands; 13, Deserts & Xeric Shrublands.

Supplementary References

1. Artés, T. *et al.* A global wildfire dataset for the analysis of fire regimes and fire behaviour. *Sci Data* **6**, 296 (2019).
2. ESA. *Land Cover CCI Product User Guide Version 2. Tech. Rep.* maps.elie.ucl.ac.be/CCI/viewer/download/ESACCI-LC-Ph2-PUGv2_2.0.pdf (2017).
3. Dinerstein, E. *et al.* An Ecoregion-Based Approach to Protecting Half the Terrestrial Realm. *Bioscience* **67**, 534–545 (2017).
